# Essay—Nerve Tissue*This paper, not being designed for publication, free use of authorities is made without special credit.

**Published:** 1874-01

**Authors:** Jas. B. Baird


### NERVE TISSUE.*

AN ESSAY READ BEFORE THE ATLANTA HISTOLOGICAL AND PATHOLOGICAL SOCIETY, ATLANTA, NOV. 15, 1873.

BY JAS. B. BAIRD, M.D.

On account of the extent of the subject, which we propse to discuss, I have thought proper to divide it, and in this paper not undertake to consider the central organs of the nervous system, except so far as they are intimately associated with the peripheric system of nerves.

The structural element of nerves, speaking generally, are of three kinds, viz: The nerve fibre, for the conduction of nerve force; the origin and the terminal organs of these fibres. We will, therefore, consider this subject systematically, under the following heads:

1. The nerve fibre.
2. The peripheric terminal organs of the nerve.
3. The centric organs of nerve fibres.

All nerve trunks are composed chiefly of nerve fibres with which are mingled connective tissue and blood-vessels. These also constitute a large portion of the central organs, forming not only the whole of the white substance, but also a considerable part of the gray matter. There are several varieties of them—the primitive nerve fibrils, representing the most simple form. They are moderately frequent in the central organs and in the neighborhood of the peripheric terminations, and are only visible by an enlargement of 500 to 800 linear. No internal structure, according to Max Schultze, can be detected in the fibres by the microscope.

A second kind of fibre is frequently met with in the central organs, distinguishable from the former only by its greater thickness. Their diameter amounts to only a few micromilimetres. These are the fibres which have been termed naked axis cylinders. The internal structure, according to the author above quoted, presents a more or less well-marked striation, resulting from the fibrous structure of the fibre, and probably to the presence of a fine granular interfibrillar material. Both the primative fibrils

* This paper, not being designed for publication, free use of authorities is made without special credit.

and the fibrillar fasciculi, as the second kind are called, may become invested with a medullary sheath, and thus present a third kind of nerve fibre—the medullated. It will be noted that the medullated fibre consists of a cortex of medullary substance, and an axis fibre or axis cylinder, which may be composed either of a single fibril or a fasciculus of fibrils. The medullary investment composed of an oily substance containing protagon, which powerfully refracts light and gives to the nerve fibres their characteristic strongly defined dark borders, forms a layer more or less thick around the axis cylinder. Notwithstanding the softness of the medullated fibres, and the absence of a protecting sheath in the nerve centers, they are preserved from injury by an "extremely delicate tenacious spongy connective substance" in which they are imbedded. The medullated fibres of the peripheric nerves, however, possess, external to the medullary layer, an additional investment of connective tissue, called the sheath of Schwann. The fibres of the optic and acoustic nerves perhaps form an exception to this rule. Schultze says of this investment, that it is either a structureless, perfectly transparent, delicate membrane, agreeing in its consistence and chemical constitution with the sarcolema of muscular fibre, or consists of several layers of fibrillar connective tissue, and as in this, presents nuclei scattered through its substance.

The strongly refractive external border of the medullary sheath prevents the feebly refractive sheath of Schwann from being seen, but the resistance and firmness of the fibres are greatly augmented by it. It also prevents the imbibition of fluids and the formation of varicosities which gives the medullated fibres destitute of this protecting sheath so characteristic an appearance under the microscope.

A fourth kind of fibre that occurs among the peripheric nerves differs from the last described only in the absence of the medullary sheath, and is hence called non-medullated nerve fibre.

These consist of a bundle of primitive fibrils, bound together by a nucleated sheath of Schwann—all of the branches of the olfactory nerve in the mucous membrane of the nose of the vertebrata, together with some of the fibres of the sympathetic, and the nerves of the greater number of invertebrate animals are composed of this sort of fibre.

Remak first discovered them among the branches of the sympathetic, distributed to the intestines of Ruminants, hence the non-medullated sympathetic fibres are known as Remak's fibres.

Remak called them ganglionic fibres.

To resume. We have, according to the preceding account—

1. Primitive fibrils.
2. Fasciculi of primitive fibrils.
3. Primitive fibrils with medullary sheath.
4. Fasciculi of primitive fibrils with medullary sheath.
5. Fasciculi of primitive fibrils invested by a sheath of Schwann.
6. Fasciculi of primitive fibrils with a medullary sheath and a sheath of Schwann.

Nos. 1 and 2 may be designated as naked axis cylinder, and, when invested by a sheath of Schwann, as simply axis cylinders.

It has not yet been decided whether nerve-fibres exist possessing a medullary sheath, a sheath of Schwann, the axis cylinder of which is composed of a single primitive fibril.

Nerve fibres, according to the foregoing, may also be divided into medullary and non-medullated, as follows:

*I. Non-medullated.*

1. Primitive fibrils.
2. Fasciculi of primitive fibrils.
3. Fasciculi of primitive fibrils with sheath of Schwann.

*II. Medullated.*

1. Primitive fibril with medullary sheath.
2. Fasciculi of primitive fibrils with medullary sheath.
3. Fasciculi of primitive fibrils with medullary sheath and sheath of Schwann.

Max Schultze, after systematizing the different forms of fibres as above, remarks that, "We see then that the primitive fibril forms the elementary constituent of all nerve fibres." This, however, according to Stricker, is contrary to the generally accepted opinion, which does not regard the primitive fibrils as the ultimate structural elements of the nerve fibres. It will be observed that the variations in the nerve fibres are dependent upon the number of fibrils united together, and the presence or absence of the sheath of Schwann, and the medullary sheath. The complicated structure of one of the medullated fibres is apparent when it is remembered that it consists of a fasciculus of primitive fibrils united together by an interfibrillar material, forming the axis cylinders and two investing sheaths. The division of the nerve fibres is a peculiar feature presented by them. It is to be observed in the nerve centers, occasionally in the nerve trunks, and frequently near their peripheric extremity. It may occur in all kinds of nerve fibres, except the primitive fibrils, according to Schultze.

Branched and ramified fasciculi of primitive fibrils are composed of the processes of many multipolar ganglion cells. Repeated sub-division of the olfactory nerve may be seen with the sheath of Schwann prolonged upon the branches.

The mode, however, most frequently described is that represented by the division of medullated fibres distributed to muscle. This manner of division is described as usually dichotomous, and includes all of the constituents of the nerve fibre. The division of the fibrillar axis cylinder probably consists merely in the gradual separation of the associated primitive fibrils. This mode of division of the axis cylinder would seem to be required for the proper performance of its physiological function. The sheaths of the medullary substance and of Schwann divide with the branches, and are finally lost at their extremities. The sum of the diameters of the branches exceed that of the trunk, and as we know that the axis cylinder diminishes in size, it follows that the sheaths must become proportionately thicker. Instead of a dichotomous division, three or four, or even twenty or thirty branches may suddenly arise from a single trunk fibre—a remarkable example of this mode of division is furnished by the electrical eel (Malapterurus electricus). Bilharz states that each of the two electrical organs which lie like masses of bacon fat beneath the skin, receives a single medullated nerve fibre, having a diameter of $\frac{1}{40}$ of a milimetre, from the medulla oblongata, which, in order that it may give a peripheric terminal fibre to each electrical plate, must divide millions of times!

It is proper to remark that the axis cylinder does not always divide into the finest nerve fibrils, but an axis cylinder of appreciable diameter, so far as is at present known, terminates without breaking up into the finest filaments. All of the nerves of special sense seem to divide into primitive fibrils at the peripheric termination, and it is stated that this is especially the case where a great variety of impressions occur within a very limited space. In these instances each fibril is provided with a peculiar terminal organ, which the limits of this paper will not allow to be described in detail. In a recent essay on this subject Max

Schultze says, in substance, as follows: "In the nasal mucous membrane fusiform easily alterable cells are found. These possess a centric and a peripheric process. The former exactly resembles the primitive nerve fibrils of the olfactory nerves, but the latter either ends at the level of the free surface of the epithelial cells, as in many mammals and fishes, or extends beyond this surface in the form of a long, stiff hair, or of several finer hairs analogous to ciliæ, but incapable of movement.

Gustatory cells, similar to the above, exist according to Schwable and Loven in the papillæ circmumvalatæ of man and mammals. Similar conditions are found in the auditory organs. In those parts where the nerve termination is simple, after leaving their medullary sheath the nerve fibres, according to Schultze, break up into primitive fibrils and penetrating between the epithelial cells become continuous with peculiar ciliated auditory cells. The optic nerve in the retina terminates in the so-called layer of rods and cones and "nucleated external granules," the last like the terminal apparatus of the olfactory nerve appear as fusiform cells with a centric and peripheric process. The centric process of the rods is a single primitive fibril, of the cones a fasciculus of fibrils. (Stricker.)

The tactile nerves of the skin terminate in what are called tactile corpuscles. These are spherical, very soft bodies, occupying the interior of many tactile papillæ of the skin. The exact mode of termination of the primitive fibrils within these bodies has not been determined. The primitive fibrils being the axis cylinder of the medullated fibre with which each of the corpuscles is provided.

The corpuscles of Vater or Pacini may be regarded as the terminal organs of the sensory nerves. These are found, in man on the plantar and volar nerves, in the subcutaneous connective tissue on the sides of the fingers and toes. On nerves supplying joints, and those lying between various muscles of the trunk and extremities. In animals they are found in many other parts of the body. Like the tactile corpuscles each of these bodies receives a medullated nerve fibre.

The corpuscle itself is described as consisting of many concentrically arranged layers of connective tissue surrounding a cavity filled with a soft, abundantly nucleated material into which the axis cylinder penetrates and terminates in a little bulb. The medullary sheath and the sheath of Schwann become continuous with the investing sheath of the corpuscle. Krause has described numerous terminal nerve corpuscles existing in the conjunctiva, genitals and other positions of the body, and differing from the Pacinian corpuscles only in the absence of the thick laminated investment.

In regard to the termination of nerves in glands Pfluger has shown that in the salivary gland, either the cells themselves or their nuclei form the terminal organ. Hensen has described the cutaneous nerves of the frog as terminating in the nuclei of the cells of the epidermis. Where there is more than one nucleus the terminal fibres are bifurcated.

In all organs, or parts of organs, in the composition of which muscular tissue plays an important role, except in some particular instances, according to J. Arnold, a similar arrangement of nerves is to be found.

In 1840 Doyere discovered that the nerve applied itself to the muscular fibre by means of a conical enlargment.

W. Kuhne, in an essay recently on the mode of termination of nerve fibre in muscle, remarks that: "The admission of loop-like extremities in the muscle can only be regarded as an expression of ignorance." The same author says: "In all transversely striated muscles the nerves terminate beneath the sarcolema, the sheath of Schwann becoming continuous with the latter. Up to this point the axis cylinder is accompanied by the medullary sheath, which is here suddenly lost. The extremity of the axis cylinder always corresponds to a remarkably broad expansion, which constantly forms a flat, branching mass. This terminal nerve plate sometimes presents the character of a membrane, and at others resembles a system of fibres. In the greater number of cases the plate rests upon a substratum of nuclei and finely granular protoplasm, whilst in others this material is absent, and the nerve plates possess the so-called terminal nerve bulbs. The extremity of the nerve never penetrates into the interior of the contractile cylinder, and, on the other hand, never entirely invests it. Short muscular fibres usually receive only one nerve; but long fibres have several.

Kuhne adds, hypothetically, that the substratum represents the remains of a formative material important in the development of both the muscular and nervous tissue, and that a similar explanation may he offered of the terminal nerve bulbs in respect to the nervous tissue

The only point that now remains to be considered is the mode of origin of the nerve fibres in the nerve centres. The precise mode of the centric origin of nerve fibres has not yet been thoroughly solved, though the microscopic examination of the ganglia of the brain, spinal cord and sympathetic nerve, shows that the cells are to be regarded as an essential part of these structures. Most of these cells are spheroidal, yet they are often very irregular in outline. They are destitute of any doubly contoured investing membrane, though each cell occupies a kind of capsule composed of nucleated connective tissue.

The majority, perhaps all of these cells possess processes. These processes, as was first observed by Remack in the vertebrata are nerve fibres. The cells are designated according to the number of processes they possess, and are denominated unipolar, bipolar or multipolar.

The spinal ganglia of man are more complex, and according to the researches, more particularly of Deiters, no matter how numerous its processes may be, a cell only gives off one peripherically coursing axis cylinder. This runs without branching, obtains sooner or later a medullary sheath, and enters one of the roots of a spinal nerve. It possesses a fibrillar structure both in the sensory and motor and ganglion cells. The other processes of these ganglion cells branch in an arborescent manner soon after their origin. Their structure is also fibrillar, but they contain a greater quantity of inter-fiibrillar granular substance than the axis cylinder. The course of the fibrils from these processes within the cell is very complicated. They pass into the cell substance in a divergent manner in every direction, and are there lost in a "confused whorl of decussating filaments." The nucleus of the cells lies imbedded in a finely granular fibrillated material, or gives the impression of a sharply defined outline. Its substance is homogeneous, and it contains a large nucleolus which stands out in bold relief as a strongly refractive spherical body. Max Schultze remarks that we may regard such a ganglion cell, from which a peripherically directed nerve fibre proceeds, as representing the source and origin of this axis cylinder, but only in the sense that the fibrils which compose the axis cylinder are collected into a group from the arborescent processes of the cell, and thus the fibres which are seen traversing the substance of the ganglion cell do not originate in the cell, but only undergo a kind of arrangement in it and then pass to the axis cylinder process or extend into the other branched processes.

Schultze goes on to say that the central origin of a single nerve fibre has never been proven, but reasoning from analogy we may conclude that they originate either from the cell substance of the nerve cell, the nucleus or the nucleolus. He even goes so far as to say that it is even conceivable, according to his observations, that there is no actual termination of the fibrils in the brain or spinal cord. In other words, that all fibrils originate at the periphery, and thus only traverse the ganglion cells.

